# Transglutaminase 2 in human diseases

**DOI:** 10.1051/bmdcn/2017070315

**Published:** 2017-08-25

**Authors:** Zsuzsa Szondy, Ilma Korponay-Szabó, Robert Király, Zsolt Sarang, Gregory J. Tsay

**Affiliations:** 1 Dental Biochemistry, Department of Biochemistry and Molecular Biology, University of Debrecen Debrecen 4010 Hungary; 2 Department of Pediatrics and Biochemistry and Molecular Biology, University of Debrecen Debrecen 4010 Hungary; 3 Celiac Disease Center, Heim Pál Children’s Hospital Budapest 1089 Hungary; 4 Department of Biochemistry and Molecular Biology, University of Debrecen Debrecen 4010 Hungary; 5 Division of Immunology and Rheumatology, Department of Internal Medicine, China Medical University Hospital Taichung 404 Taiwan; 6 School of medicine, College of Medicine, China Medical University Taichung 404 Taiwan

**Keywords:** Transglutaminase, Inflammation, Cancer, Fibrosis, Cardiovascular Disease, Neurodegenerative Disease, Celiac Disease

## Abstract

Transglutaminase 2 (TG2) is an inducible transamidating acyltransferase that catalyzes Ca(2+)-dependent protein modifications. In addition to being an enzyme, TG2 also serves as a G protein for several seven transmembrane receptors and acts as a co-receptor for integrin β1 and β3 integrins distinguishing it from other members of the transglutaminase family. TG2 is ubiquitously expressed in almost all cell types and all cell compartments, and is also present on the cell surface and gets secreted to the extracellular matrix *via* non-classical mechanisms. TG2 has been associated with various human diseases including inflammation, cancer, fibrosis, cardiovascular disease, neurodegenerative diseases, celiac disease in which it plays either a protective role, or contributes to the pathogenesis. Thus modulating the biological activities of TG2 in these diseases will have a therapeutic value.

## 1. Introduction

Transglutaminases (TG) belong to a family of structurally and functionally related enzymes that catalyse Ca^2+^-dependent post-translational modifications of proteins by introducing proteinprotein cross-links, amine incorporation, and site-specific deamidation [1, 2]. In humans, nine members of the TG family have been identified, out of which eight are catalytically active. TG2 is the most studied, multi-functional member of the transglutaminase family, and is very unique among them, because besides being a transglutaminase it also possesses GTPase, protein disulphide isomerase and protein kinase enzymatic activities (reviewed in ref.3). TG2 is expressed in almost all cell compartments such as the cytoplasm, mitochondria, recycling endosomes, and nucleus. It is also present on the cell surface and gets secreted to the extracellular matrix *via* non-classical mechanisms [4]. The structure of TG2 contains four domains: N-terminal β-sandwich domain, catalytic core domain, and two C-terminal β-barrel 1 and β-barrel 2 domains. The protein can exist both in a closed (in the presence of GTP) and in an opened active conformation when Ca^2^+ is bound to the enzyme [5]. TG2 has a conserved 3D structure and catalytic triad shared by other family members [6], but also other unique protein sequences, very often intrinsically disordered regions and short linear motifs that make the protein an ideal protein-protein interaction partner. Thus, TG2 has a fi-bronectin interaction site, a syndecan-4 site and an MFG-E8 site which participate in cell adhesion, migration and phagocytosis, α1-adrenoceptor and PLCδ1 sites involved in intracellular signalling, and a BH3 domain that couples the protein to apoptosis regulation [7]. Increasing evidence indicate that these and many other non-enzymatic interactions play physiological roles and enable diverse TG2 functions in various protein networks in a context-specific manner [8, 9] explaining why TG2 appears as a mediator in so many human diseases. Interestingly, published exome sequencing data from various populations have not uncovered individuals with homozygous loss-of-function variants for TG2. Thus it seems that TG2 is under purifying selection not allowing generation of even heterozygous common variants [10]. These genetic data indicate an essential, may be so far uncovered novel role for TG2 in the human organism.

## 2. Transglutaminase 2 in fibroproliferative diseases

Fibroproliferative diseases, including progressive kidney disease, pulmonary fibroses, systemic sclerosis, liver cirrhosis and cardiovascular disease are a leading cause of morbidity and mortality and can affect all tissues and organ systems. Fibrosis is a wound-healing response to chronic stimuli that has gone out of control [11]. Under healthy conditions following injury, a regeneration program is initiated, which involves activated T lymphocytes that produce profibrotic cytokines such as transforming growth factor (TGF)-β and interleukin (IL)-13 [12, 13], and activated B lymphocytes that produce IL-6 [14]. These cytokines activate both macrophages and fibroblasts. As a result, activated fibroblasts transform into α-SMA-expressing collagen producing myofibroblasts. In addition, α-SMA-expressing myofibroblasts can derive also from the bone marrow (fibrocytes) [15], as well as from epithelial cells which underwent epithelial-mesenchymal transition (EMT) [16]. The development of fibrosis is associated with aberrant repair, persistence of collagen deposition, and vascular remodeling, and all these events are driven by an enhanced uncontrolled myofibroblast activity [17, 18]. TGF-β can not only augment EMT and the production of interstitial collagens, fibronectin, and proteoglycans by myofibroblasts [19], but it can also trigger its own production by myofibroblasts, thereby establishing an autocrine cycle of myofibroblast differentiation and activation that characterizes fibroproliferative diseases. Enhanced TGF-β production was found in patients suffering from idiopathic hypertrophic cardiomyopathy [20], renal fibrosis [21] or liver cirrhosis [22], while mice overexpressing active TGF-β1 developed diseases characterized by fibrosis, such as progressive cardiac hypertrophy [23] or hepatic injury [24]. Thus it is generally accepted that active TGF-β plays a central role in driving fibroproliferative diseases.

There are several ways through which TG2 can promote tissue fibrosis. First of all, TG2 and the production of active TGF-β are strongly linked. TGFβ is secreted in a latent form, non-covalently bound to its cleaved propeptide which is disulphide linked to latent TGF-β binding protein (LTBP) family proteins which assist in its folding, secretion and localization, and allow mechanical activation of the cytokine [25]. The N-terminus of LTBPs has been shown to be a substrate for TG2 which promotes their covalent incorporation into the extracellular matrix [26]. In addition, TG2 was found to contribute to the activation of macrophage-derived TGF-β [27], and to promote TGF-β1 transcription [28].

Besides being linked to active TGF-β formation, TG2 is profibrotic also, because it can cross-link several matrix proteins making them more resistant to protein breakdown [29]. In fact, TG2, and not lysyl oxidase, dominates the early calcium-dependent remodeling of fibroblast-populated collagen lattices during wound healing [30]. In addition, in the cytosol the G protein function of the enzyme has effects on the cell survival [31]. As an integrin coreceptor, TG2 enhances cell adhesion and motility [32, 33] activates cell survival pathways that can operate in myofibroblasts as well [34, 35], and promotes phagocytosis of dead cells by macrophages [36, 37]. Both activated macrophages and fibroblasts express elevated levels of TG2 in a metastatic tumor antigen 1 (MTA1)-dependent manner [38].

Interestingly, not only TG2 is required for proper TGF-β formation, but TGF-β itself promotes the transcription of TG2. Thus, TG2 contains TGF-β response elements both in its promoter [39] and in two of its enhancers [40]. As a result, TGF-β drives TG2 expression, while TG2 contributes to the transcription, secretion and activation of TGF-β leading to the formation of an additional level of self-amplification loop in the pathogenesis of fibrosis. Not surprisingly, fibroproliferative diseases are characterized not only by enhanced TGF-β production, but also by enhanced TG2 expression [41, 42]. The central role of TG2 in maintaining these diseases is proven by the observation that TG2 knock out mice are protected from fibrosis in several experimental fibrosis models [43, 44]. Based on these observations TG2 activity was inhibited both in a pulmonary and in a renal experimental model of fibrosis, and inhibition of the enzyme significantly reduced the development of fibrosis in both fibrosis models [45, 46]. Thus it is concluded that inhibition of extracellular TG2 activity might be beneficial in the treatment of fibrotic diseases.

## 3. Transglutaminase 2 and cancer

Another group of diseases, in which inhibition of TG2 might be beneficial, is cancerous diseases. Recent studies indicate that cancer cells express elevated levels of TG2, and elevated TG2 levels are associated with an aggressive cancer phenotype and drug resistance in most of these tumors [47]. Moreover, TG2 levels are especially enhanced in the cancer stem cells, and TG2 is required for their survival, migration and invasion [48]. Thus a correlation between elevated cancer TG2 levels and cancer aggressiveness was reported in the case of colorectal [49], breast [50], pancreatic [51], ovarian [52], esophageal squamous cell [53] cancer, glioblastomas [54], malignant melanomas [55], renal [56] and cervical squamous cell carcinomas [57] and hepatocellular carcinomas [58]. In addition, TG2 was found to be a biomarker of cervical intraepithelial neoplasia [59].

Though several mechanisms have been reported through which TG2 promotes cancer survival, tumor progression and invasion, many of these effects are attributed to the extracellularly located TG2. TG2, acting as a protein crosslinking enzyme, can modify the structure and stability of extracellular matrix (ECM) in a way that it supports integrin-dependent ECM binding and migration of cancer cells [29]. Extracellular TG2 can crosslink S100A4 promoting metastasis [60]. TG2 acts also as an integrin co-receptor for the β_1_, β_3_, β_4_, and β_5_ integrins, and facilitates integrin-mediated signaling pathways [33], which partly promote the growth factor signaling pathway, thus promote cell growth [61, 62], partly activate the PI3K/AKT mediated-cell survival pathway leading to inhibition of both apoptosis [63] and autophagic cell death [64]. In addition to promoting the integrin signaling pathway as a co-receptor, TG2 was also shown to enhance the activity of the PI3K signaling pathway by directly forming a complex with PI3K and src [65] and by downregulating PTEN [66], a negative regulator of the PI3K pathway. TG2 can inhibit apoptosis in cancer cells also by directly inhibiting caspase-3 activity *via* forming a crosslinked multimer, or by upregulating NF-κB activity, which transcribes anti-apoptotic proteins [67]. NF-κB driven IL-6 production in breast cancer cells was also shown to contribute to the aggressiveness of the tumor [68]. Though TG2 was shown to crosslink IκB [69], in cancer cells either extracellular TG2 induces the noncanonical pathway of NF-κB activation by activating IκB kinase [70], or cytoplasmic TG2 directly interacts with IκB [71], which might be affected by a PKA-dependent serine-216 phosphorylation of TG2 [67].

In addition to these mechanisms, it was found for renal cell carcinoma that TG2 can compete with human doubles minute 2 homolog (HDM2) for binding p53, and facilitate the degradation of p53 *via* the autophagosomal system [72].

Epithelial-mesenchymal transition (EMT) is a developmentally regulated process in which adherent epithelial cells lose their epithelial characteristics and acquire mesenchymal properties, including fibroid morphology, characteristic changes in gene expression and increased invasion and resistance to chemotherapy [73]. Increasing body of evidence indicates that acquisition of EMT by cancer cells is an important mechanism in the progression and pathogenesis of cancer, and TG2 promotes EMT in his closed form [74–79]. The mechanism involves activation of the PI3K and NF-κB signaling pathways and inhibition of GSK3β [78]. The effect of TG2 on cancer cells might be effected also by the fact that different splicing variants of TG2 might be expressed in healthy and malignant cells [80].

It seems that chronic inflammation that strongly predisposes for cancer formation [81], and hypoxia [82] which characterizes the fast growing cancer cells are the two main driving forces that lead to overexpression of TG2 in cancer cells, since TG2 expression is directly regulated by those pro-inflammatory cytokines that activate NF-κB, by TGF-β, and by the hypoxia activated HIFs. Interestingly, TG2-expressing cells display high basal levels of HIF-1α expression even under normoxic conditions, and suppression of either TG2 or NF-κB (p65/RelA) reduces HIF-1α level. Chromatin immunoprecipitation studies revealed that TG2 forms a complex with p65/RelA and that the complex binds to the NF-κB binding site in the HIF-1α promoter [71]. Thus in cancer cells an autoregulatory loop exits, in which NF-κB and HIF-1α upregulate the expression of TG2, while TG2 further enhances the NF-κB and HIF-1α-driven transcription including its own transcription resulting in the maintenance of high TG2 levels.

## 4. Transglutaminase 2 in cardiovascular diseases

Cardiovascular diseases (CVDs) are among the leading causes of death worldwide. CVDs include several disorders affecting the blood vessels *-e.g.* coronary heart disease, deep vein thrombosis, vascular calcification, cerebrovascular and peripheral arterial diseases- and the heart, such as rheumatic and congenital heart disease.

Atherosclerosis, affecting the blood vessels, contributes significantly to the development of myocardial infarction and ischemic stroke. It is characterized by inflammation of endothelial cells, proliferation of vascular-smooth-muscle cells, and deposition of excessive cholesterol, accumulation of apoptotic and necrotic macrophages and appearance of transformed macrophages, so called foam cells, in the arterial wall forming the atherosclerotic plaque [83]. Under normal circumstances TG2 is widely expressed in macrophages, smooth muscle cells and endothelial cells and it was reported to accumulate in plaques [84] and to interact with atherosclerotic processes in several ways. TG2 was shown to activate the NFκB pathway and promote inflammation by crosslinking the NFκB inhibitor IKB-α leading to TNF-α and nitric oxide synthase expression [69]. The promoter of TG2 contains NFκB and cytokine responsive element contributing to formation of activation loop in inflammatory macrophages [85]. In this way TG2 might facilitate initial damage of endothelial cells by promoting inflammatory response in macrophages. On the other hand, TG2 recently was described to dampen inflammation by the histaminylation of fibrinogen leading to sequestration of the pro-inflammatory histamine and to inhibition of fibrinogen binding to endothelial cells which prevents leukocyte migration to inflammatory site [86]. In contrast, using TG2 deficient macrophage murine atherosclerosis model it was shown that lack of TG2 results in increased atherosclerotic plaque formation and higher number of necrotic cells in plaques [87]. This phenomena can be explained by the reduced apoptotic cell engulfment capacity of TG2 null macrophages [36] resulting in accumulation of apoptotic cells which undergo subsequent secondary necrosis and increase plaque size.

As described above, TG2 facilitates deposition and stabilization of ECM by facilitating TGF-β activation and crosslinking of ECM, respectively [25–28]. Still, there are controversial data available about the role of TG2 in plaque stability. Using apo-E/TG2-/- murine model Van Herch and co-workers found that TG2 deficiency resulted in decreased collagen content and increased inflammation of plaques, which are features of a more unstable plaque [88]. On the contrary, in a similar model William and his co-workers found no alteration in the composition or calcification of plaques between wild type and apo-E/TG2-/- mice [89]. Vascular calcification is frequently found in atherosclerotic lesions and is general complication of long-term Vitamin K antagonist, warfarin administration. Warfarin was shown to enhance TG2 expression and activity which in turn enhances the β-catenin pathway [90]. β-catenin promotes cardiovascular calcification by enhancing differentiation of vascular smooth muscle cells and aortic valve interstitial cells into osteoblast-like cells [91]. As a result, systemic administration of a TG2 inhibitor attenuated the warfarin-induced vascular calcification supporting TG2’s role in this phenomenon [90].

TG2 is also present in blood platelets [92]. These cells play a central role in hemostasis and in the pathogenesis of thrombosis and atherosclerosis. Platelet adhesion to inflamed blood vessel is the initial trigger for the formation of either an effective hemostatic plug or of an intravascular thrombus [93]. During activation, platelets release the content of their α-granules and dense bodies to promote blood coagulation. Activated platelets bind several α-granule-released proteins including fibrinogen, von Willebrand factor, thrombospondin and fibronectin -all of them being a substrate for TG2 - and are referred as collagen- and thrombin-activated (COAT) platelets [94]. Both the release of the content of α-granules and the binding of these procoagulants happens through TG2-mediated covalent binding of serotonin to proteins called serotonylation [95, 96]. COAT platelet levels were shown to increase in patients with non-lacunar ischemic stroke and subarachnoid hemorrhage, while patients with spontaneous intracerebral hemorrhage had lower COAT platelet count compared to controls [97]. As a result, approaches considering therapeutic regulation of TG2 activity in circulation will have to consider these possible side effects.

Essential or idiopathic hypertension is the most common form of hypertension. Notably, pharmacological inhibition of TG2 by cystamine resulted in reduction in blood pressure in spontaneously hypertensive rats underlining the importance of TG2 in elevated blood pressure [98]. Recent studies linked the immune system to development of essential hypertension [99, 100]. In serum of hypertonic patients markedly elevated proinflammatory cytokine (C-reactive protein, INF, IL-1β, IL-6, and IL-17) levels can be detected leading to enhanced TG2 expression. Pro-inflammatory IL-6 and hypoxia-inducible factor-1a induced TG2 was found in mice to posttranslationally modify angiotensin receptor type 1 (ATI) resulting in the recognition of the altered receptor by immune system and in production of autoimmune antibodies. These antibodies bind to and activate AT1 inducing vasoconstriction and hypertension [101]. In addition, TG2-dependent isopeptide modification of AT1 was shown to stabilize the expression of the receptors by preventing their ubiquitination-dependent degradation. This also led to hypertension in an experimental mouse model [102].

TG2, on the other hand plays a protective role in ischemia reperfusion injury of the heart by regulating ATP synthesis [103]. All these evidences underline the involvement of TG2 in cardiovascular diseases and indicate that modulation of TG2 activity might provide new therapeutic approaches in the treatment of these diseases as well.

## 5. Transglutaminase 2 in celiac disease and other gastroenterological diseases

In celiac disease, TG2 is a specific target of a conditional autoimmune mechanism driven by exogenous cereal peptides. In genetically predisposed individuals, ingestion of wheat, rye and barley leads to small intestinal villous atrophy, malabsorption and the production of antibodies against TG2. Gluten peptides derived from these cereals are rich in glutamine and proline residues (especially those from the alcohol-soluble gliadin fraction of gluten) and are good substrates for the transamidating enzyme reaction catalyzed by TG2 [104]. In addition, deamidation can be a preferred reaction outcome in the usual conditions of the stomach and gastrointestinal tract (acidic pH, relatively low concentrations of amine acceptor substrates). Deamidation in the pattern characteristic for TG2 (Q-X-P motifs) renders gliadin peptides more immunogenic, by making them more fitting into the HLA-DQ groove of antigen presenting cells [105]. Only HLA-DQ2.5, 2.2 and DQ8 can present gliadin peptides to T cells and celiac disease occurs only in persons with these genetic alleles [106]. For this reason, celiac disease is common in Caucasian populations, in the Arab world and in India, while HLA-DQ2 and DQ8 molecules are rare in other African and Asian countries where celiac disease occurs only exceptionally [107]. HLA-DQ2 and DQ8 molecules require acidic residues in certain positions for peptide docking and the effective binding of gliadin-specific T cells to occur [108]. Activation of CD4+ T cells leads to inflammation by the production of cytokines, mainly interferon-gamma. Further inflammatory signals are derived from the activation of innate immune mechanisms, including IL-15 [109–111]. Due to the high amount of proline residues, gliadin peptides, especially a 33-mer alpha-gliadin peptide containing multiple overlapping deamidated sequences, are resistant to human gastric, pancreatic and intestinal brush border proteolytic enzymes [112, 113] and thus longer peptides can be transported through the absorptive epithelial layer and can be encountered by T cells [114]. Gliadin-specific T cells as helpers are held responsible for the activation of specific sets of B cells producing antibodies against gliadin peptides and also against TG2. Most commonly, production of TG2-specific antibodies is explained by the hapten-carrier hypothesis [115] driven by gliadin-specific T cells providing help to TG2-specific B cells, but certain molecular mimicry between gliadin peptides and TG2 can also occur [116, 117]. In any case, the autoimmune reaction to TG2 only runs in the presence of gliadin peptides and stops when the patient is placed on a gluten-free diet [118]. A glutenfree diet also leads to the reversion of all disease manifestation (provided they are still of reversible nature) and constitutes an effective treatment for celiac disease.

**Fig. 1:**
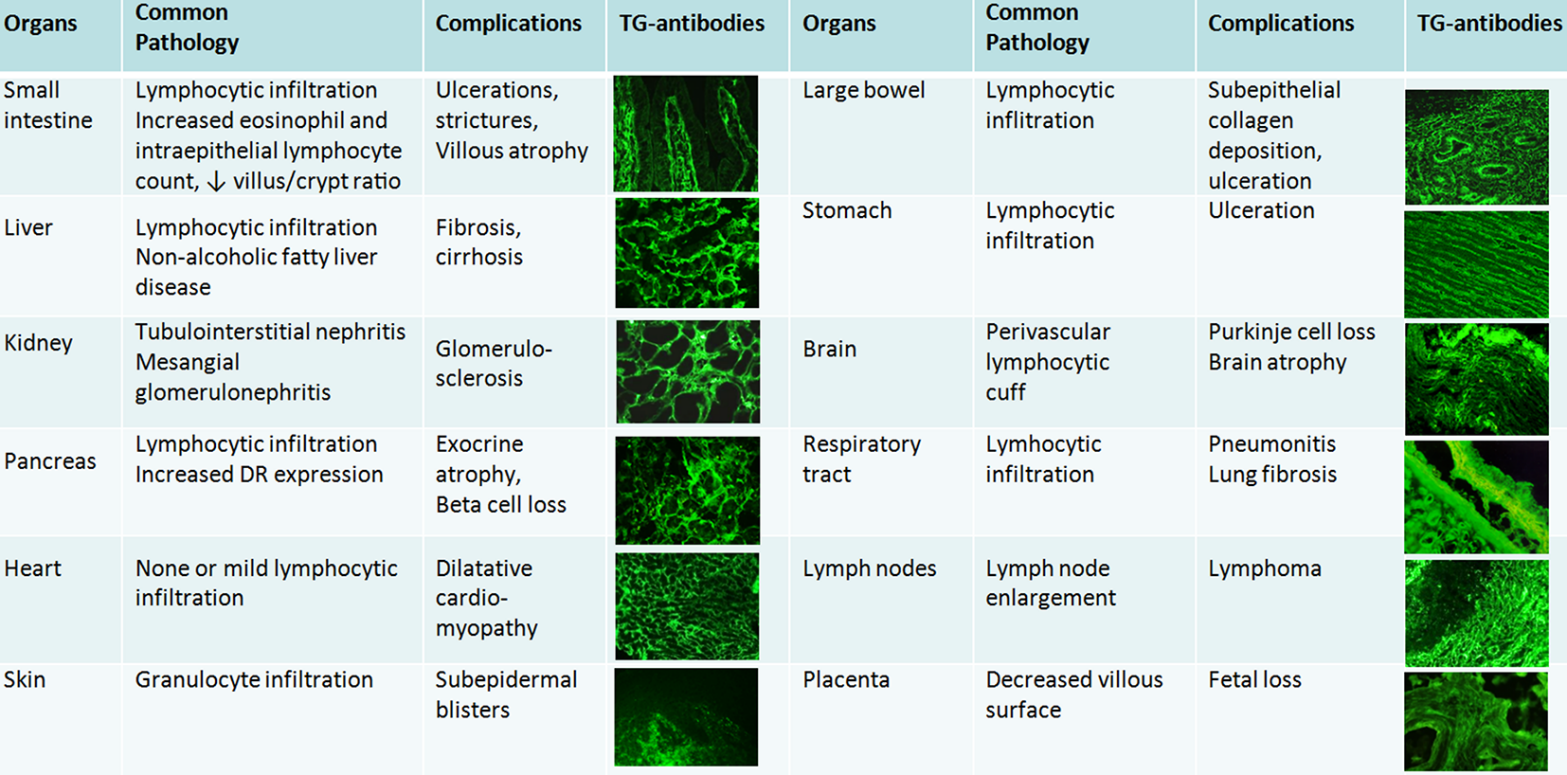
Overview of system manifestations in celiac disease. The common denominator is the deposition of autoantibodies on the surface of extracellular TG2 (or in the skin TG3) shown here by immunofluorescence (green).

TG2-specific antibodies are produced in all celiac disease patients and they target the same few TG2 epitopes [119, 120], so they inherently belong to the disease process. Although in up to 10% of patients TG2 antibodies cannot be detected from serum, the TG2 autoantibodies are found deposited and bound to tissue TG2 also in these subjects [121]. Experimental data and clinical observations suggest that these antibodies are biologically active and possibly contribute to disease manifestations [122–125]. Histological lesions seen by conventional stainings constitute of inflammation and non-specific organ changes. Small bowel villous flattening and atrophy with the elongation of crypts is a non-specific reaction of the bowel to injury [126] also seen in a number of other, non-celiac disease conditions including bacterial and viral (Rotavirus, HIV-1) infections, graft-versus host disease, irradiation or drug-induced *(e.g.* olmesartan) enteropathy [127]. The only celiac-specific component is the production and tissue binding to TG2 of autoantibodies along reticulin fibers [128], endomysium and vessel structures [129] both in the intestine as well as in extraintestinal sites (Fig. 1), where it has great diagnostic value, provided a frozen specimen is available for immunofluorescent studies. Commonly seen extraintestinal manifestations of celiac disease involve almost all organs, including liver, heart, kidney, pancreas, brain and placenta [130, 131]. Celiac disease is thus regarded today as a systemic autoimmune disorder [129, 131], and not only a malabsorptive intestinal disease. Some of the produced antibodies also may target TG3 (in the skin) or TG6 (in the brain), in addition to TG2 [132, 133].

Anti-TG2 antibodies are exceptionally good disease activity biomarkers in medicine [134] and by their detection even symptom-free patients can be identified among family members or in the general population. Rapid immuno-chromatographic point-of-care tests are now widely available for this purpose [135]. Initially, celiac antibodies were detected by indirect immunofluorescent method when incubating patient serum on normal tissue sections. The resulting binding patterns (called in the 20th century endomysial [EMA], reticulin [ARA] or anti-jejunal antibodies [JEA]), are exclusively TG2-dependent [136] and thus EMA, ARA and JEA represent celiac-specific TG2 autoantibodies against extracellular TG2 epitopes [137]. High level of circulating anti-TG2 antibodies (above 10x of upper limit in ELISA detection), confirmed also by positive serum EMA result in patients with malabsorptive symptoms and HLA-DQ2 or DQ8 background reliably predict villous atrophy [134, 137] and thus can be used according to new European diagnostic guidelines [131] as substitute for histology assessment. Decline of antibodies on a gluten-free diet occurs and whenever they remain positive for more than 1-2 years, it indicates dietary transgressions, thus anti-TG2 measurements are also used with success as follow-up evaluations in everyday practice [137].

Although the lifelong gluten-free diet is currently the medical choice of treatment for celiac disease, therapy adherence may be as low as 50% in some countries. Therefore, future research should focus on alternative treatment options [138]. Degradation of ingested gluten in the gastrointestinal tract by bacterial enzymes, inhibitors of TG’s deamidating activity, tightening of tight junctions, cytokine inhibitors, blocking of DQ2-mediated presentation, among others, may provide some therapeutic benefit, but clinical utility of these approaches remains to be proven before they can be suggested to patients.

TG2-targeted antibodies may be produced also in other autoimmune or inflammatory conditions, including inflammatory bowel diseases, but epitope and IgG subclass usage differs from celiac disease [139] and clinical implication of these antibodies is uncertain. In addition, in a mouse colitis model TG2 was reported to be required for survival [140]. Involvement of TG2 has been described in cystic fibrosis as well, where it may contribute to TGFβ1 activation and signaling and induction of epithelialmesenchymal transition [141]. TG2 inhibitors may thus have a role in the stabilization of the epithelium and decrease of inflammation also in this disorder.

## 6. Transglutaminase 2 and other inflammatory diseases

Efficient execution of apoptotic cell death followed by efficient clearance mediated by professional and by nonprofessional neighboring phagocytes, is a key mechanism in maintaining tissue homeostasis. TG2 is expressed by both apoptotic cells and by their engulfing macrophages. In the context of the apopto-phagocytosis program TG2 is anti-inflammatory because in the apoptotic cells TG2 promotes apoptosis, once the program is initiated [142], prevents the release of the pro-inflammatory cell content by crosslinking the proteins of apoptotic cells [143], contributes to the formation of a „find me” signal which acts as a chemoattractant for macrophages [144], and accelerates the cell surface appearance of phosphatidylserine, the main recognition signal for the engulfing macrophages [145]. In macrophages acting as an integrin β3 coreceptor TG2 is required for proper migration toward the apoptotic cells [32] and for the opening of the engulfing portal, thus for proper phagocytosis of apoptotic cells [36, 37]. TG2 also contributes to the activation of latent TGF-β [146] that acts as an anti-inflammatory molecule in this context [147]. Not surprisingly, in the absence of TG2 the normally silent clearance of apoptotic cells is associated with inflammation and development of an SLE-like autoimmune disease in mice [36].

Improper clearance of apoptotic neutrophils explains partly also the phenomenon that in TG2 null mice the resolution of inflammation in gouty arthritis is delayed [148]. Gouty arthritis is a characteristically intense acute inflammatory reaction which is initiated by precipitation of monosodium urate crystals that are taken up by tissue resident macrophages. These phagocytes activate the NLRP3 inflammasome, resulting in the activation of caspase-1 and processing and secretion of interleukin-1β (IL-1β) which drive the inflammation. In addition to contributing to the clearance of dying neutrophils, it was also shown that the metastatic tumor antigen 1- TG2 pathway regulates the production of TGF-β1 in macrophages which opposes the MSU crystal-induced JAK2-dependent pro-inflammatory cytokine formation of IL-1β [149]. In line with these findings TG2 expression was found to be up-regulated in the synovium tissue and synovial fluid mononuclear cells from patients with gouty arthritis [149]. In addition, it was also shown that the ribosomal protein S19 (RP S19) polymer cross-linked at Lys122 and Gln137 by transglutaminases released from apoptotic neutrophils acts as a C5aR ligand during the resolution phase of acute inflammation. In the absence of TG2 activity the resolution was also found to be delayed in the carrageenan-induced acute pleurisy in C57BL/6J model mice [150].

Interestingly, TG2 does not play an anti-inflammatory role in every pathological context. For example, TG2 null mice are protected against lipopolysaccharide (LPS)-induced endotoxic shock [151]. In BV-2 microglia cells, TG2 promotes the LPS-induced pro-inflammatory response by crosslinking IκB, an inhibitor of NF-κB transcription factor that drives the transcription of several pro-inflammatoy genes [69]. In endothelial cells, TG2 promotes phosphorylation of RelA/p65 at Ser536, a crucial event that confers transcriptional competency to the DNA-bound NF-κB. As a result, a marked reduction in ICAM-1 expression and lung neutrophil sequestration was observed in TG2 knockout compared to wild type mice after intraperitoneal LPS challenge [152]. Accordingly, transglutaminase inhibitors ameliorate endotoxin-induced uveitis [153] and trapping TG2 by a fusion protein attenuates corneal inflammation and neovascularization [154].

TG2 also drives the all-trans-retinoic acid-induced differentiation syndrome [155] characterized by unexplained fever, acute respiratory distress with interstitial pulmonary infiltrates, and/or a vascular capillary leak syndrome leading to acute renal failure in patients with acute promyelocytic leukemia. TG2 expression was also found to be increased in the skin biopsy of patients with psoriasis, a chronic autoimmune skin disorder characterized by hyperproliferation of the keratinocytes in the epidermis, though no correlation between TG2 expression levels and the disease duration, stage of disease and subtype of psoriasis could be found [156]. Mast cell derived TG2 was reported to participate in the pathogenesis of chronic urticaria [157]. TG2 also contributes to experimental multiple sclerosis pathogenesis and clinical outcome by promoting macrophage migration [158]. TG2 was also shown to contribute to the development of collagen-induced arthritis, an experimental model of rheumatoid arthritis, by posttranslationally modifying the immunodominant T-cell epitope [159] and by facilitating invadopodia formation and cartilage breakdown [160]. In line with these findings TG2 was found to be a biomarker of osteoarthritis [161]. In addition, TG2 was suggested to be a biomarker for idiopathic inflammatory myopathies as well [162].

## 7. Transglutaminase 2 in neurological disorders

Neurodegenerative diseases, such as Alzheimer’s disease, Parkinson’s disease, supranuclear palsy, Huntington’s disease and other polyglutamine diseases, are characterized in part by aberrant cerebral transglutaminase activity and by accumulation and deposition of cross-linked proteins in affected brains [163–168]. Although these aggregates are composed of specific proteins characteristic of the respective neurodegenerative disease, the tendency of these proteins to self-interact and form toxic aggregates seems to be a common phenomenon in these diseases. It has been shown that the accumulating proteins, such as amyloid-beta, tau, a-synuclein or huntingtin are all substrates of TG2 [169], and proteomic analysis of the cross-linked proteins in the brain from these diseases confirmed that indeed TG2 could be involved in their formation [164]. Though, conclusive experimental findings about the role of TG2 in the development of these human diseases have not yet been obtained, results obtained from animal models of these diseases indicate that inhibition of TG2 activity might have a therapeutic value in preventing the formation of cross-linked proteins [170].

## 8. Possibilities to modulate transglutaminase 2 functions with the aim of affecting the pathogenesis of transglutaminase-linked diseases

The fact that the involvement of TG2 in various pathological conditions was clearly demonstrated makes TG2 a potential therapeutic target, raising up the need for TG2 modulators, inhibitors. There are different strategies to downregulate TG2 activity. Inhibitors can switch off transglutaminase activity, while other compounds decrease the expression or modulate the trafficking of TG2. This session surveys some of the possibilities to block TG2 functions. These tools have already been applied in research to reveal physiological and pathological roles of TG2, and point toward potential therapeutic possibilities.

The classical approach is to inhibit the catalytic activity of the enzyme. Based on their mechanism of action, TG2 inhibitors form three major groups: competitive amines, reversible and irreversible inhibitors [171–173]. The first applied TG2 inhibitors were amines, for instance cadaverine-derivatives, putrescine, which compete with biogenic amine or lysine donor protein substrates preventing the formation of naturally occurring isopeptide crosslink. Superficially, this group contains cystamine, a special disulphide diamine, with multiple inhibitory mechanism and offtarget effects like inhibition of caspase-3 [171]. Cysteamine, the reduced form of cystamine, which is an approved drug to treat cystinosis, also inhibits TG2, but probably by the formation of a mixed disulphide with the catalytic cysteine residue [171]. In biological systems amines have no specific TG2 inhibitory potential which forced the discovery of more specific reversible inhibitors.

One group of reversible inhibitors are non-hydrolysable GTP analogues and mimics that stabilize the inactive, closed conformation of the enzyme. Interestingly, Mehta and co-workers patented small compounds targeting the GTP binding pocket of TG2 by in silico screening based on their docking scores [174]. Another source of reversible inhibitors was found by experimental screening of small compound libraries [175] and then the design of new ones on structural similarity with the hits from the screening [176]. Recently it was published that acylidene-oxoindoles, one group of the reversible inhibitors, target a Ca^2+^-binding site and at sub-saturating Ca^2^+ concentration, surprisingly, they can act as an agonist of TG2 activity [177]. Due to pharmaceutical safety the application of reversible inhibitors would be desirable, however, their relatively low solubility and efficiency make difficult to reach the effective therapeutic concentration [172].

Development of selective irreversible inhibitors significantly increased the specificity and efficiency of inhibition making these compounds beneficial for therapy. Irreversible inhibitors generally target the active site cysteine residue, where the nucleophile thiol group forms stable bond with the electrophilic functional group of the inhibitor. First, iodoacetamide was applied for irreversible alkylation of Cys277 in TG2, because halogens are good leaving groups, but instead it reacted with other surface localized nucleophile residues. To get a more specific inhibitor, the general 3-halo-4, 5-dihydroisoxazol structure of acivicin, a glutamine analogue inhibitor of gamma-glutamyl transpeptidase and glutamine amidotransferase and transglutaminases [178], was combined with an aromatic side chain and N-terminal carbamoyl group based on Cbz-PheGln dipeptide substrate. These changes with the replacement of chloride to bromide resulted in KCC009 [179] that was tested in various disease models in rat and mouse *in vivo* experiments. KCC009 treatment of glioblastoma and meningioma cells resulted in enhanced apoptosis and sensitivity to chemotherapy through impairment of TG2-dependent fibronectin organization and cell survival signaling acting as a chemo sensitizing agent [180, 181]. KCC009 treatment also sensitized lung and ovarian cancer cells for TRAIL and cisplatin induced apoptosis, respectively [182, 183]. KCC009 was efficient to block warfarin-induced osteogenic vascular calcification in a rat model [184]. It also decreased macrophage immigration in the central nervous system in rat chronic-relapsing experimental autoimmune encephalomyelitis offering promising approach to treat multiple sclerosis [185]. In these animal experiments KCC009 had low toxicity, good oral availability but its short serum half-life and low solubility, slightly higher than its Ki value, are its disadvantages [171, 177].

ERW1041E was developed by the modification of KCC009 based on the TG2 preferred gluten derived peptide substrate to generate a small compound for celiac disease treatment [186]. It blocs *in vivo* the intestinal activation of TG2 in a C57BL/6J mouse model after intraperitoneal injection of polyinosinic-polycytidylic acid, providing the first evidence for TG2 inhibition in mammalian intestine [187]. However, due to the cross-reactivity of ERW1041E and other dihydroisoxazole inhibitors with other transglutaminase isoforms, some modifications have been made for developing of more efficient compounds with higher potency and selectivity [188]. The resulting improved dihydroisoxazoles seem to be very promising drugs for celiac disease treatment to prevent the TG2-dependent formation of highly immunogenic deamidated gliadin peptides.

Michael acceptor inhibitors cover the α, β-unsaturated carbonyl compounds that participate in addition reaction with thiol nucleophiles in many active site cysteine containing enzymes. One of the widely used members of this group is NC9. It has acrylamide warhead developed with PEG spacer and dansyl group, which decreased its affinity and efficiency. However, applying it as a biological probe provided reasonable benefits [173]. NC9 was applied to study conformational changes of TG2 and recently a study claimed that it can reduce cancer stem cell survival [189, 190]. In this group of molecules several further compounds were developed characterised by Keillor’s group, CHDI Foundation and Zedira [191–194]. Z-DON is a very selective inhibitor of TG2 produced by replacing the Michael acceptor warhead with 6-diazo-5-oxo-L-norleucine (DON) [195]. In addition, the gluten based peptide sequence modified DON warhead made the crystallization of TG2 possible providing hard evidence about its conformational change during activation [196].

Inhibitors with imidazolium-based and sulfonium warhead were also designed based on the Cbz-PheGln backbone linked them with various length spacer to increase their hydrophilic property [197, 198]. These compounds inhibit angiogenesis and are patented for the treatment of eye diseases like, age-related macular degeneration and diabetic retinopathy [174].

A new direction in the blockage of TG2 functions is the application of antibodies or peptides. On the cell surface TG2 regulates adhesion and migration interacting with fibronectin, integrins, syndecan-4 heparane-sulphates [199]. To prevent metastasis formation peptide and antigen binding antibodies were patented, which cover the heparan-sulphate binding site negatively influencing the adhesion and migratory potential of the cells [199]. In 2015 lysine containing cell-permeable peptides were patented with picomolar Ki values for the treatment of disorders with high transglutaminase activity, while other peptides were patented to prevent polymerisation of IκB and the consequent activation of NF-κB pathway [174]. Antibodies also can modulate TG2 activities in a very specific and efficient way. Recently it was confirmed that some coeliac antibodies can stabilise TG2 in an active conformation resulting in enhanced TG2 activity [200], while other antibodies have inhibitory effect on TG2, and some of these are patented for the treatment of liver, kidney fibrosis, and diabetic nephropathy [174, 201]. Interestingly, in an Alzheimer disease model [202] and in pancreatic cancer cells [203] downregulation of TG2 expresssion by curcumin and rottlerin, respectively, could provide therapeutic possibility.

So far, there is only one TG2 inhibitor in clinical trial (phase1b). Zedira (Germany) has developed ZED1227, a small pyridinon derivative, for the treatment of coeliac disease for blocking the TG2-mediated deamidation of gliadin peptides. Interestingly, Zedira has also developed ZED3197, a peptidomimetic based drug candidate to target blood coagulation factor XIII, another member of the transglutaminase family, as an anticoagulant without prolonged bleeding time [174]. Finally, better understanding of the transglutaminase related pathomechanisms and the increasing structural knowledge about TG2 provide promising future to discover therapeutically applicable drugs against progressive diseases mediated by transglutaminases.
